# *In Vivo* Imaging of Retinal Hypoxia in a Model of Oxygen-Induced Retinopathy

**DOI:** 10.1038/srep31011

**Published:** 2016-08-05

**Authors:** Md. Imam Uddin, Stephanie M. Evans, Jason R. Craft, Megan E. Capozzi, Gary W. McCollum, Rong Yang, Lawrence J. Marnett, Md. Jashim Uddin, Ashwath Jayagopal, John S. Penn

**Affiliations:** 1 Department of Ophthalmology and Visual Sciences, Vanderbilt University School of Medicine, Nashville, TN, USA; 2 Department of Molecular Physiology and Biophysics, Vanderbilt University School of Medicine, Nashville, TN, USA; 3 A. B. Hancock, Jr., Memorial Laboratory for Cancer Research, Departments of Biochemistry, Chemistry and Pharmacology, Vanderbilt Institute of Chemical Biology, Center for Molecular Toxicology and Vanderbilt-Ingram Cancer Center, Vanderbilt University School of Medicine, Nashville, TN, USA; 4 Pharma Research and Early Development, Roche Innovation Center Basel, F. Hoffmann-La Roche, Ltd., Basel, Switzerland

## Abstract

Ischemia-induced hypoxia elicits retinal neovascularization and is a major component of several blinding retinopathies such as retinopathy of prematurity (ROP), diabetic retinopathy (DR) and retinal vein occlusion (RVO). Currently, noninvasive imaging techniques capable of detecting and monitoring retinal hypoxia in living systems do not exist. Such techniques would greatly clarify the role of hypoxia in experimental and human retinal neovascular pathogenesis. In this study, we developed and characterized HYPOX-4, a fluorescence-imaging probe capable of detecting retinal-hypoxia in living animals. HYPOX-4 dependent *in vivo* and *ex vivo* imaging of hypoxia was tested in a mouse model of oxygen-induced retinopathy (OIR). Predicted patterns of retinal hypoxia were imaged by HYPOX-4 dependent fluorescence activity in this animal model. In retinal cells and mouse retinal tissue, pimonidazole-adduct immunostaining confirmed the hypoxia selectivity of HYPOX-4. HYPOX-4 had no effect on retinal cell proliferation as indicated by BrdU assay and exhibited no acute toxicity in retinal tissue as indicated by TUNEL assay and electroretinography (ERG) analysis. Therefore, HYPOX-4 could potentially serve as the basis for *in vivo* fluorescence-based hypoxia-imaging techniques, providing a tool for investigators to understand the pathogenesis of ischemic retinopathies and for physicians to address unmet clinical needs.

Retinopathy of prematurity (ROP)^1^,^2^, proliferative diabetic retinopathy (PDR)^3^ and retinal vein occlusion (RVO)^4^ are blinding conditions with neovascular components that develop from ischemia-induced retinal hypoxia. In ROP, ischemia arises from attenuated physiologic blood vessel development in preterm infants receiving supplemental oxygen to compensate for under-developed lung function^5^,^6^. When the oxygen therapy is discontinued and the infant is placed in normoxia, the peripheral retina is avascular (ischemia), and becomes hypoxic^7^. Hyperglycemia and hyperlipidemia are causally linked to capillary dropout and vasoregression in the diabetic retina, leading to focal avascularity (ischemia) and incipient retinal hypoxia that triggers the onset of PDR. Although the exact etiology of branch RVO is unknown, arteriolar compression at an arteriovenous crossing may lead to the formation of an occlusive thrombus in the affected vein, reducing blood flow (ischemia) and presumably initiating the development of retinal hypoxia^8^. Retinal hypoxia activates the transcription of hypoxia-regulated pro-angiogenic growth factors/cytokines such as vascular endothelial cell growth factors (VEGF)^9^ and angiopoietin-like protein-4 (ANGPTL4)^10^. These factors elicit a neovascular response that manifests in the formation of preretinal neovascular structures that enhance morbidity, often leading to blindness in individuals afflicted with ROP, PDR and RVO. Based on these forgoing considerations, the ability to detect, quantify, track and image retinal hypoxia becomes of paramount importance, as it would allow a better understanding of the pathophysiology of ischemic retinopathies. Furthermore, early detection of hypoxia before the onset of any overt neural or vascular anomalies, could perhaps aid the physician to assess whether prophylactic therapy is indicated. Finally, hypoxia imaging and quantification would be useful in terms of gauging the efficacy of therapy on established disease.

Several analytical platforms have been applied to measuring retinal oxygen pressure (pO_2_) levels including, but not limited to, the use of oxygen sensitive electrodes^11^, nuclear magnetic resonance (NMR)^12^, retinal oximetry^13^, oxygen-dependent molecular phosphorescence quenching^14^, doppler optical coherence tomography (D-OCT)^15^, visible-light optical coherence tomography (vis-OCT)^16^ and immunohistochemical analysis^17^. Oxygen electrodes permit the acquisition of reliable data but are invasive and are extremely difficult to use in rodents due to their smaller globes. NMR is minimally invasive, however it is not a direct measure of oxygen pressure and the resolution is appreciably less than optical methods^18^,^19^,^20^. Retinal oximetry and doppler OCT are methods that rely on the differences in the spectral characteristics of oxyhemoglobin and hemoglobin in the intravascular compartment, and their relative abundance in arteries compared to veins. These measurements may be performed in living systems, however, they are indirect and mathematical modeling is required to estimate the perivascular oxygen pressure. Phosphorescent quenching relies on intravascular oxygen levels providing only limited assessment of the oxygen pressure in the retinal tissue. Pimonidazole-mediated immunohistochemistry is a common method used to determine retinal hypoxia, but the technique is limited by its exclusively *ex vivo* method of examination^21^,^22^. Though a number of methods have been reported in the literature to visualize tumor hypoxia using positron emission tomography, none have been applied to the detection of retinal hypoxia. Furthermore, they carry the risks associated with use of short-lived isotopes. For these and other reasons, the techniques discussed above are not optimal for measuring retinal hypoxia in living animals in real time.

Previously, we described the development of HYPOX-1, HYPOX-2 and HYPOX-3 as sensitive fluorophore-labeled imaging probes to detect hypoxia^23^,^24^. These fluorescent probes are reduced by nitroreductases or azoreductases, facilitating their retention within hypoxic cells of the retina, allowing *ex vivo* hypoxia detection^25^. However, their application to *in vivo* imaging was limited due to poor pharmacokinetic parameters. To achieve the goal of *in vivo* imaging, we continued our efforts to synthesize and characterize a probe having the correct pharmacokinetic properties to allow enhanced diffusion into capillary-free retinal tissue. To address these needs, we synthesized HYPOX-4 and tested its ability to discriminate between normoxia and hypoxia *in vivo,* by hypoxia-induced fluorescence enhancement. We tested HYPOX-4 in mouse OIR, a preclinical model of ischemia-induced retinopathy in which retinal hypoxia is a critical pathologic component. Herein we report the results.

## Materials and Methods

### Reagents, equipment and cells

Low glucose DMEM, DMEM/F12, Fetal Bovine Serum (FBS), GlutaMAX, Gentamicin/Amphotericin B and Penicillin-Streptomycin were obtained from GIBCO; Grand Island, NY. The human retinal pigment epithelial cell line, ARPE-19 was purchased from ATCC; Manassas, VA. The human Müller cell line (MIO-M1) was kindly provided by Dr. G. A. Limb (Moorfields Institute of Ophthalmology, London, UK)^26^. The rat retinal neuronal cell line R28 was purchased from KeraFast; Boston, MA. A humidified cell culture chamber with a ProOx 110 oxygen control device was obtained from BioSpherix Inc.; Parish, NY. A Hypoxyprobe immunodetection kit (anti-pimonidazole-adduct antibody) was purchased from Hypoxyprobe Inc.; Burlington, MA. The secondary anti-rabbit IgG conjugated to Alexa Fluor® 647 (AF647), Prolong Gold mounting media with DAPI and Alexa Fluor® 488- or 647-conjugated isolectin B4 were purchased from Life Technologies; Grand Island, NY.

### Mice

C57BL/6J timed-pregnant dams were purchased from Charles River Laboratories; Chicago, Illinois. All animal procedures used in this study were approved by the Vanderbilt University Institutional Animal Care and Use Committee and were performed in accordance with the ARVO Statement for the Use of Animals in Ophthalmic and Vision Research.

### Imaging of retinal hypoxia in mouse OIR

In order to produce OIR, mouse pups and their dams were placed in 75% oxygen from P7 to P12^27^. On P12, litters were removed to room air and after two hours, HYPOX-4 (60 mg/kg in PBS) was administered to mouse pups by intraperitoneal injection. Twenty-four hours post-injection, *in vivo* HYPOX-4 dependent fluorescence imaging was performed. Briefly, mice were anesthetized with ketamine/xylazine, eyes were dilated with 1% tropicamide, and placed on a warm platform; fluorescent and bright field fundus images were acquired using the Micron IV retinal-imaging system (Phoenix Research Laboratories; Pleasanton, CA). Then, *ex vivo* imaging of HYPOX-4 dependent fluorescence was performed. The mice were sacrificed, enucleated and the globes were fixed in 10% neutral buffered formalin (NBF). Retinas were dissected and stained directly with Alexa Fluor® 647-conjugated isolectin B4, then flat-mounted on a microscope slide with Prolong Gold mounting medium (Life Technologies, Grand Island, NY). Images were captured using an epifluorescence Nikon Eclipse T*i*-E inverted microscope (Melville, NY).

Some mice received intraperitoneal injections of pimonidazole hydrochloride at a concentration of 60 mg/kg body weight two hours after removal to room air; they were sacrificed one hour later and enucleated. The globes were fixed in 10% NBF for two hours; retinas were dissected and washed with tris-buffered saline (TBS); then they were blocked/permeabilized in 10% donkey serum with 1% Triton X-100 and 0.05% Tween 20 in TBS for 6 hours and stained with rabbit antibody against pimonidazole-adducts followed by the secondary anti-rabbit IgG conjugated to Alexa Fluor® 647- and Alexa Fluor® 488-conjugated isolectin B4. The retinas were mounted on microscope slides with Prolong Gold mounting medium. Images were captured using an epifluorescence Nikon Eclipse T*i*-E inverted microscope (Melville, NY).

### Retinal cell culture

ARPE-19 cells were cultured in DMEM/F12 supplemented with 10% FBS, 1X GlutaMAX, and 1X Gentamicin/Amphotericin B. MIO-M1 and R28 cells were cultured in low glucose DMEM supplemented with 10% FBS, 1X GlutaMAX, and 1X Penicillin-Streptomycin. Cells were maintained in a humidified environment with 5% CO_2_ at 37 °C unless otherwise noted. The cells were cultured in 96 well plates or 8 well chamber slides and treated with HYPOX-4 concentrations ranging from 10 to 100 μM in complete medium containing supplemental nutrients. To establish a hypoxic environment, a calibrated proOx sensor (BioSpherix Inc.; Parish, NY) was used to maintain lower oxygen pressures. The oxygen level was monitored using the Traceable^TM^ Dissolved Oxygen Meter Pen (Fisher Scientific; Pittsburgh, PA). To confirm induction of hypoxia, cells were cultured on chamber slides and treated with 100 μM pimonidazole hydrochloride diluted in complete medium, then subjected to hypoxia or normoxia for 4 hours and immunostained for pimonidazole-adducts according to manufacturer’s protocol.

### *In vitro* hypoxia-induced, HYPOX-4 dependent fluorescence assay

ARPE-19 and MIO-M1 cells were seeded at 20,000 and R28 at 15,000 cells per well, respectively, in a clear bottom 96-well black plate. When cells were 80% confluent, they were treated with 100 μM HYPOX-4 unless otherwise specified, in complete medium and incubated in normoxia or hypoxia for 4 hours. The cells were washed with pre-warmed Hank’s Buffered Salt Solution (HBSS). Fluorescence intensity was measured (Absorbance: 490 nm, Emission: 520 nm) using a microplate reader (Biotek; Winooski, VT).

### *In vitro* imaging of retinal cells using HYPOX-4

R28 cells were seeded at a density of 45,000 cells per well in 4-well chamber slides. When cells were 90% confluent, cells were treated with HYPOX-4 in complete medium, or with pimonidazole hydrochloride diluted in complete medium. The cells were cultured in normoxia or hypoxia for 4 hours. Cells were washed 3 times with HBSS, fixed with 10% NBF for 10 minutes at room temperature, washed 3 times with TBS and mounted with Prolong Gold with DAPI mounting media. Pimonidazole-adducts were immunostained according to manufacturer’s protocol. HYPOX-4 dependent fluorescence images were captured using the epifluorescence microscope.

### Electroretinography (ERG) measurements

ERG measurements were performed according to previously published methods^28^,^29^. Briefly, ERG analysis was performed on mice injected with HYPOX-4 (100 mg/kg) at one week post-injection. Animals were dark adapted overnight, anesthetized with ketamine/xylazine, dilated with 1% tropicamide, and placed on a warm platform within the Ganzfeld dome of a Diagnosys LLC Espion Electrophysiology system (Lowell, MA, USA). Mice were exposed to flashes of light ranging from −4 to 2 log cd.s/m^2^ and the amplitudes of *a*-wave and *b*-wave were measured from baseline to peak. The amplitude of the *a*-wave and *b*-wave were plotted as a function of luminance.

### TUNEL assay

TUNEL assays were performed using Click-iT *in situ* apoptosis detection kit (Life Technology, USA). Adult C57BL/6J mice were treated with HYPOX-4 (100 mg/kg); they were sacrificed 24 hour later and enucleated. The eyes were fresh frozen in 30% sucrose and embedded in TissueTec OCT for cryosectioning (7 μm sections). The retinal transverse sections were then stained for fragmented DNA by incorporating alkyne-modified EdUTP nucleotide followed by detection with Alexa Fluor® 647 azide in apoptotic cells. Retinal transverse sections from C57BL/6J mice that did not receive HYPOX-4 were treated with DNase I and stained as a positive control. Counts of TUNEL + nuclei were performed by counting total number of stained nuclei across 400 μm sections of each individual sample.

### Cell proliferation assay

Retinal cell-lines were treated with HYPOX-4 (100 μM) and proliferation was measured using the bromodeoxyUridine (BrdU) incorporation assay according to the manufacturers protocol. Briefly, retinal cells were seeded in a 96-well plate, cultured for twenty four hours. Then, cells were treated with HYPOX-4 or vehicle controls diluted in complete media and allowed to incubate for 24 h. Four hours prior to the end of the incubation, BrdU was added at a concentration of 10 μM and incorporation of BrdU was quantified by ELISA with the BrdU cell proliferation ELISA kit (Exalpha Biologicals; Shirley, MA) according to manufacturer’s protocol.

### Statistics

Data are presented as mean ± SD. Student’s t tests were performed to compare 2 samples and, for comparison of more than 2 samples, one-way ANOVA was performed using Prism 6 (Graph- Pad, San Diego, CA). p ≤ 0.05 was considered as statistically significant.

### Synthesis of HYPOX-4

The synthesis and related characterization data for HYPOX-4 is supplied in the supplementary information (SI).

## Results

### *In vitro* hypoxia-induced HYPOX-4 fluorescence activity

**Figure 1:**
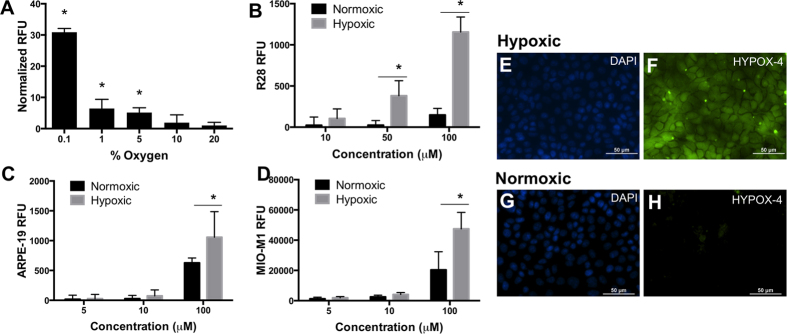
Sensitivity and hypoxia-specificity of HYPOX-4 in retinal cells. (**A**) R28 cells were treated with HYPOX-4 (100 μM) and variable oxygen concentrations. HYPOX-4 dependent fluorescence increased with decreasing oxygen concentration. (**B**) R28, (**C**) ARPE19 and (**D**) MIO-M1 cells were treated with concentrations of HYPOX-4 ranging from 10 to 100 μM and 0.1% oxygen concentration; a HYPOX-4 dose-dependent fluorescence was observed in all of these cell types. (**E,F**) R28 cells were treated with HYPOX-4 (100 μM) and 0.1% oxygen for 4 hours. Hypoxia-specific fluorescence cell imaging was achieved. (**G,H**) Minimal fluorescence was observed in normoxic cells (n = 8, *p < 0.05).

Cultures of rat retinal neuronal cells (R28) were treated with 100 μM HYPOX-4 and exposed to variable oxygen concentrations ranging from 0.1% to 20.9% for 4 hours. HYPOX-4 dependent fluorescence was maximal at 0.1% oxygen (Fig. 1A). Next, using R28 cells (Fig. 1B), retinal pigment epithelial cells (ARPE-19, Fig. 1C) and human Müller cells (MIO-M1, Fig. 1D), the oxygen concentration was maintained at 0.1% and the HYPOX-4 concentration was varied from 10 μM to 100 μM. HYPOX-4 dependent fluorersecence was observed with highest signal to noise ratios at the100 μM dose, for all cell types. Additionally, we also observed increased fluorescence in the normoxic cells at the highest concentration (100 μM) that we applied. This may be due to baseline azoreductase activity causing increased retention of HYPOX-4 that becomes more significant at the highest concentration. Finally, we performed *in vitro* cellular imaging using R28 cells. Hypoxia-specific, HYPOX-4 dependent fluorescence activity facilitated hypoxia imaging in this cell-line, as shown in Fig. 1E,F. The punctate bright green fluorescence was consistently obseved in these R28 cultures using pimonidazole and several other hypoxia sensing probes, and also the R28 cell line is known to be a heterogeneous cell population^23^. Therefore, this fluorescence may represent cell-associated hypoxic signal. Minimal HYPOX-4 dependent fluorescence was observed in normoxic cells (Fig. 1G,H). Hypoxia was confirmed in these cell lines by immunostaining pimonidazole-adducts (Figure S5)^24^. All *in vitro* experiments were replicated a minimum of three times. Where appropriate, results were statistically evaluated by ANOVA.

### *In vivo* imaging of retinal hypoxia in the mouse model of oxygen induced retinopathy (OIR)

**Figure 2:**
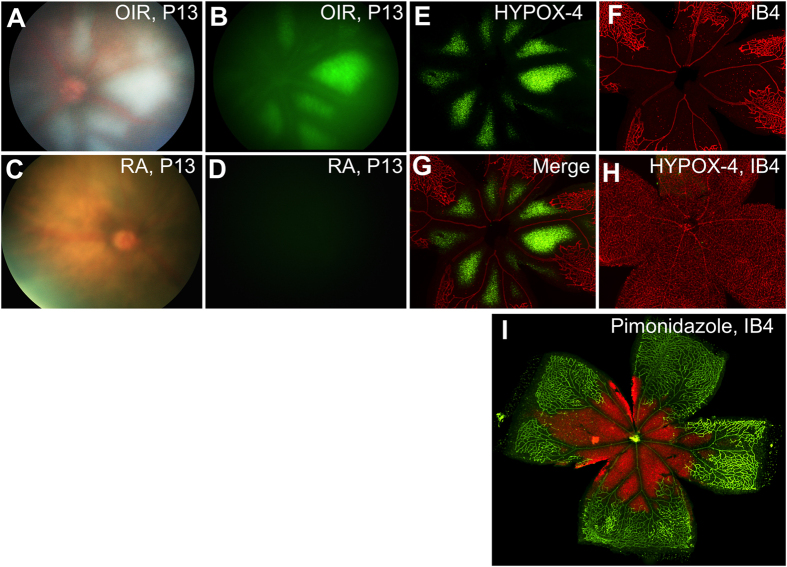
*In vivo* imaging of retinal hypoxia in mouse OIR (P13) and age matched room air (RA) pups. HYPOX-4 was administered systemically to OIR mouse pups 2 hours after return to room air on P12, as well as to age-matched room air pups. *In vivo* imaging was performed 24 hours post-injection of HYPOX-4. (**A**) Bright field image of OIR (P13) retina; (**B**) An image of the same retina *in vivo*, hypoxia was clearly detected by HYPOX-4 dependent fluorescence within the central avascular retina (green); (**C**) Bright field image of age-matched RA pup (P13); (**D**) HYPOX-4 dependent fluorescence was undetectable in aged-matched RA pups; (**E**) OIR mouse retina showing *ex vivo* HYPOX-4 dependent fluorescence in the central avascular retina (green); (**F**) The same retina counterstained with IB4, highlighting the peripheral vascular retina; (**G**) E and F merged; (**H**) RA pups showed minimal *ex vivo* HYPOX-4 dependent fluorescence; IB4 staining of the retinal vasculature (red) from an RA pup; (**I**) Hypoxia was confirmed in OIR (P12) pups by immunostaining of pimonidazole-adducts (red); blood vessels were counterstained with IB4 (green).

*In vivo* imaging of retinal hypoxia was performed in OIR mice^21^. HYPOX-4 was administered by intraperitoneal injection 2 hours after mice were returned from oxygen exposure chambers to room air (P12); age-matched room air (RA) control pups were similarly treated. *In vivo* fluorescence imaging was performed 24 hours post HYPOX-4 injection. HYPOX-4 dependent fluorescence was observed and it was localized to the central avascular retina where the capillary bed was attenuated (Fig. 2). *In vivo* and *ex vivo* HYPOX-4 dependent fluorescence was undetectable in age matched RA (P13) control pups. Retinal hypoxia in OIR pups was confirmed by *ex vivo* pimonidazole-adduct immunostaining. The *en face* images were highly similar to those obtained by *in vivo* HYPOX-4 dependent fluorescence (Fig. 2I), both overlapping with regions of retinal avascularity. These results indicate that HYPOX-4 may be used to reliably assess and image retinal hypoxia in a living system in real time.

### *Ex vivo* HYPOX-4 dependent fluorescence imaging of retinal transverse sections

**Figure 3:**
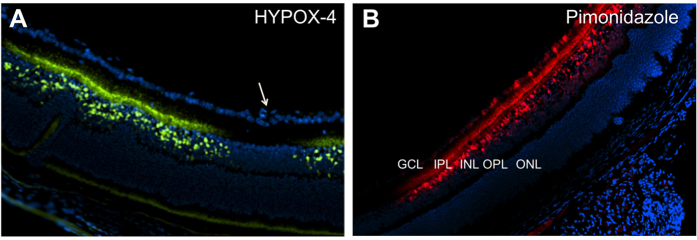
Localization of HYPOX-4 and pimonidazole in transverse retinal sections from OIR mice. OIR pups (P12) were treated with HYPOX-4 or pimonidazole and the spatial distribution of hypoxia was determined in retinal cross-sections. (**A**) HYPOX-4 dependent fluorescence activity indicated alternating regions of hypoxia in the inner retina overlapping with retinal avascularity (green); hypoxia was visualized in the inner plexiform and inner nuclear layers. Presumably, oxygen diffusion out of the major vessel indicated by the white arrow, inhibits increased azo/nitroreductase activities and consequently the retention of HYPOX-4. (**B**) Pimonidazole-adduct immunostaining confirmed retinal hypoxia in the inner plexiform and inner nuclear layers; additionally, this method detected hypoxia in the ganglion cell layer (red). (**A,B**) retinal nuclei were stained with DAPI (blue). Abbreviations: GCL = ganglion cell layer, IPL = inner plexiform layer, INL = inner nuclear layer, OPL = outer plexiform layer, ONL = outer nuclear layer.

*Ex vivo* fluorescence imaging of transverse retinal sections from OIR pups treated with HYPOX-4 was performed. HYPOX-4 dependent fluorescence indicated laterally alternating regions of hypoxia in the inner retina (Fig. 3A). Within a hypoxic region, proceeding from the inner limiting membrane (ILM) in a sclerad direction, hypoxia was observed in the inner plexiform and inner nuclear layers. Hypoxia was not observed sclerad to the inner nuclear layer. The presence of hypoxia in the inner retina was confirmed using pimonidazole-adduct immunostaining in transverse OIR retinal sections (Fig. 3B). The *ex vivo* patterns of retinal hypoxia obtained confirmed hypoxia in the inner plexiform and inner nuclear layers. The pimonidazole method also detected hypoxia in the retinal ganglion cell layer.

### Toxicity of HYPOX-4

**Figure 4:**
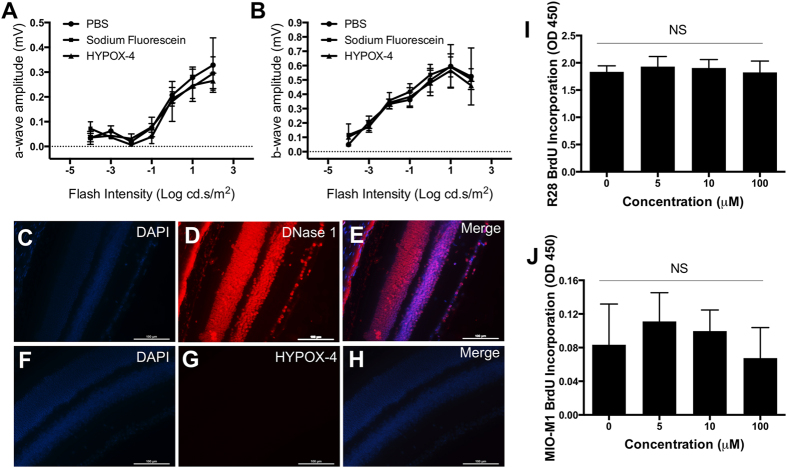
Effect of HYPOX-4 on retinal physiology was assayed in RA-raised mice via electroretinography (ERG) analysis. (**A,B**) ERG measurements of dark-adapted mice 7 days post systemic administration of HYPOX-4 revealed no significant changes in mean *a*-wave and *b*-wave amplitudes at various flash intensities compared to vehicle (PBS) and sodium fluorescein (control) groups. The TUNEL assay was performed in retinal transverse sections to assess retinal apoptosis and taken as a measure of retinal toxicity. RA mice were treated with 100 μM HYPOX-4 or DNase 1; HYPOX-4 showed no cellular apoptosis. (**C,F**) DAPI staining of nuclei; (**D**) DNase 1-treated retinal transverse sections serving as a positive control, fragmented DNA was clearly visible; (**G**) HYPOX-4 treated retinal transverse sections showed no cellular apoptosis; (**E**) C and D merged; (**H**) F and G merged. (**I,J**) *In vitro* cellular proliferation was assessed by the BrdU assay using HYPOX-4 treated R28 and MIO-M1 cells. No effect on cellular proliferation was observed.

*In vivo* toxicity was assessed by electroretinography (ERG) measurements in retinas from RA mice. HYPOX-4 probe (100 mg/kg) was injected systemically, and ERG measurements were recorded in dark-adapted mice seven days post-administration. No significant changes in mean *a*-wave and *b*-wave amplitudes at various flash intensities were observed as compared to vehicle-treated mice (Fig. 4A,B). *Ex vivo* analysis of transverse retinal sections from RA mice treated with HYPOX-4 was performed to detect retinal cell apoptosis using the TUNEL assay. No apoptosis was observed, indicating no acute toxicity as compared to the positive control retinal tissues (Fig. 4C–H). Cell proliferation assays were performed in R28 and MIO-M1 retinal cells treated with variable concentration of HYPOX-4 ranging from 0–100 μM; BrdU incorporation was analyzed. HYPOX-4 had no effect on cell proliferation (Fig. 4I,J). The ERG experiments were replicated three times. The results were statistically evaluated by ANOVA for mean *a*-wave and *b*-wave amplitudes at various flash intensities in different treatments groups. The cell proliferation assays were replicated three times, and were evaluated by Student’s *t*-test for statistical significance.

## Discussion

The synthetic strategy of HYPOX-4 was predicated on the need for a molecule with hypoxia-sensitive functionality, superior hypoxia-induced fluorescence and pharmacokinetic properties that would allow optimal tissue diffusion and bioavailablity, all required for *in vivo* imaging. Oregon Green dye has several advantages for its application to biological systems. It has: a high extinction coefficient, high fluorescence quantum yield, pH insensitivity in the physiological range, high photostability and good tissue penetration. The 2-nitroimidazole moiety was incorporated into the structure of HYPOX-4 because it is reduced by nitroreductase, an enzyme activity that is increased in tissue hypoxia, allowing 2-nitroimidazole to yield hypoxia-sensitive functionality. The solubility of HYPOX-4 was compared directly with the clinically relevant pimonidazole hydrochloride, using the octanol-water partition coefficient measurement method. Similar to pimonidazole hydrochloride, HYPOX-4 is highly soluble in aqueous medium in the free base form (Figure S3, Table SI 1). HYPOX-4 also possesses high photostability at room temperature in solution for at least 20 hours as determined by kinetic fluorescence studies (Figure S6).

We tested the hypoxia-dependent fluorescence of HYPOX-4 in ARPE-19, MIO-M1 and R28 cells (Fig. 1). We found that HYPOX-4 was efficiently internalized by these cells and an enhanced fluorescence reporter activity was observed in hypoxic cells at ≤5% (pO_2_ ≤ 38 mmHg) as compared to normoxic controls. We confirmed that hypoxia was achieved in these retinal cell lines by pimonidazole-adduct immunostaining (Figure S5)^24^. These *in vitro* studies provided evidence for the feasibility of HYPOX-4 to report the hypoxic condition in a living system, warranting the extension of these studies to the *in vivo* setting using a rodent model of ischemic retinopathy. HYPOX-4 was tested in cell proliferation assays and had no effect on cell proliferation (Fig. 4I,J).

We performed *in vivo* experiments to determine whether hypoxia-dependent HYPOX-4 fluorescence could be observed in living animals predisposed to ischemic retinopathy. We tested HYPOX-4 in an established mouse model of oxygen-induced retinopathy (OIR)^27^. In this model, OIR mice are exposed to 75% oxygen for five days from P7 to P12, causing vaso-attenuation, resulting in a central avascular retina. On P12, the mice are placed in normoxia, and *ex vivo* pimonidazole-adduct immunostaining experiments indicate the central avascular retina becomes hypoxic within a few hours^21^,^30^,^31^. We systemically administered HYPOX-4 to OIR mice 2 hours after return to room air (P12) and to age-matched normoxic controls. After 24 hours post-HYPOX-4 administration, *in vivo* imaging clearly demonstrated a HYPOX-4 dependent fluorescence within the central avascular retina, and this fluorescence was absent in adjacent perfused retinal regions. This observation clearly indicates hypoxia in the central avascular retina (Fig. 2). *Ex vivo* evaluation of HYPOX-4 fluorescence from the same retinas was in close agreement with the *in vivo* findings, and IB4 counterstaining confirmed localization of hypoxia to the central capillary-free regions of the retina. These results agree with similar findings we obtained using the *ex vivo* pimonidazole-adduct immunostaining technique. *In vivo* and *ex vivo* HYPOX-4 fluorescence was undetectable in age-matched RA controls after twenty-four hours of administration. In OIR retinas, the fluorescence signal was barely detectable after seventy-two hours.

In transverse retinal sections from OIR mice, HYPOX-4 dependent fluorescence indicated hypoxic cells in the inner nuclear layer with processes extending to the inner plexiform layer (Fig. 3A, Figure S4)^21^. Hypoxia was also assessed by pimonidazole-adduct immunostaining. This method confirmed hypoxia in the inner nuclear and plexiform layers (Fig. 3B); however, it was also detected in the retinal ganglion cell layer, creating a discrepancy between the results obtained by the two techniques. These results indicate that HYPOX-4 might discriminate between different levels of hypoxia in the inner nuclear layer and the retinal ganglion cell layer. In C57BL/6 OIR mice (the strain used in this study) it is plausible that there is lower oxygen pressure at the inner nuclear and plexiform layers compared to the retinal ganglion cell layer on day P12 through P13 (the first twenty four hours post oxygen exposure), corresponding to the times we assessed hypoxia, for the following reasons. The superficial vascular plexus is almost fully developed at the time oxygen treatment is initiated on P7^32^. Alternatively, the deep and intermediate vascular plexi have just begun to form, and exposure to hyperoxia suppresses their continued development. Vasoobliteration of the superficial plexi is maximal in the central retina two days post initiation of hyperoxia, and there is evidence that revascularization (superficial plexi) begins shortly thereafter^31^. Given these considerations, it is most likely that ganglion cells adjacent to any residual or regenerated superficial vascular plexus, experience higher oxygen pressures than the inner nuclear and plexiform layers in OIR mice at the time we assessed hypoxia. Furthermore, even in normal adult mice and rats with a fully developed retinal vasculature, the oxygen pressures are lower at the inner nuclear layer compared to the ganglion cell layer^33^,^34^. Additionally, it is unlikely that the pO_2_ at the RGC layer is appreciably affected by an oxygen source other than the retinal vasculature. Measurements of oxygen levels in experimental models and in normal human eyes, suggest that oxygen diffuses out of the retinal arterioles and into the vitreous where it is consumed by the nearby retinal tissues, creating an oxygen gradient that decreases from the posterior to the anterior segment of the eye^35^. In a kitten OIR model, using O_2_ microelectrodes to measure retinal pO_2_, preretinal pO_2_ levels approached 0 mmHg immediately above the avascular area, indicating that oxygen is not delivered to the RGC layer from a vitreous source in the absence of the retinal vasculature^36^,^37^. Taken together, these data suggest that the vitreous is not an independent source of oxygen that provides increased pO_2_ at the RGC layer.

Therefore, the discrepancies we observed on comparison of HYPOX-4 dependent fluorescence imaging and pimonidazole-adduct immunostaining in transverse retinal sections may be best explained by a lower oxygen pressure detection threshold for HYPOX-4 relative to pimonidazole. This explaination is further supported by our *in vitro* studies. HYPOX-4 dependent fluorescence was observed at a threshold of pO_2_ ≤ 38 mmHg in retinal cell lines cultured in hypoxia (normoxia; pO_2_ ≈ 160 mmHg); whereas pimonidazole detects hypoxia at pO_2_ < 76 mmHg. We also propose that this lower detection limit is advantageous for the detection of oxygen pressures that are functionally significant to neovascular pathogies. For example, experimental evidence indicates that hypoxia induces Müller cells and astrocytes to express and secrete increased retinal VEGF levels that promote preretinal neovascularization in experimental ischemic retinopathies. A previous study indicated that hypoxic induction of VEGF, in an immortalized Müller cell line was nominally significant at pO_2_’s ≤ 38 mmHg^38^. Accordingly, retinal pO_2_’s ranging from <76 to 38 mmHg, detectable by pimonidazole but not HYPOX-4, are not likely to be related to disease processes. In fact, it is known that oxgen pressures in the normal adult rodents fall within this range. We did not perfom retinal pO_2_ measurements in our experimental cohorts, although these data are valuble for determing the actual *in vivo* pO_2_ ranges detected by HYPOX-4. These measurements are very difficult in adult rodents and present an even more formidable challenge in P12–P18 mouse pups. However, published measurements in the normal adult mouse indicate a pO_2_ ≈ 5.0 mmHg at the inner nuclear layer^34^. Therefore, it is plausible that pO_2_ in mouse OIR pups is <5.0 mmHg at the inner nuclear layer, given that development of the deep capillary plexus is substantially retarded in this model. In our *in vitro* work, we observed maximum HYPOX-4-dependent fluorescence at <1 mmHg. Hence, it is reasonable to expect that the ranges of oxygen pressures observed *in vitro* and *in vivo* by HYPOX-4-dependent fluorescence overlap.

Electroretinography (ERG) measurements in dark-adapted RA mice seven days post systemic administration of HYPOX-4 revealed no significant changes in mean *a*-wave and *b*-wave amplitudes compared to vehicle indicating no acute effect of HYPOX-4 on retinal physiology (Fig. 4A,B). *Ex vivo* analysis of the retinal transverse sections from RA mice treated with HYPOX-4, were also examined using the TUNEL assay. No significant toxicity related to apoptosis was observed as compared to the positive control tissues (Fig. 4C–H). Furthermore, HYPOX-4 had no effect on *in vitro* cell-proliferation indicating that it would not affect retinal mitogenesis.

In summary, we have developed a facile route for the synthesis of HYPOX-4, a hypoxia-sensitive imaging agent, by conjugating 2-nitroimidazole to the fluorescent dye, Oregon green. HYPOX-4 is a novel probe that is not acutely toxic to retinal tissues and demonstrates pharmacokinetic properties required for efficient systemic delivery and bioavailabilty within the retina. Using HYPOX-4, we were able to detect retinal hypoxia *in vivo* in mouse model of OIR. To our knowledge, this is the first report of real time hypoxia imaging in living animals by a fluorescence-based method in the retina. HYPOX-4 hypoxia-induced retinal imaging is non-invasive and it promises to be an excellent tool for diagnosis and monitoring retinal hypoxia in preclinical disease models and patients.

## Additional Information

**How to cite this article**: Uddin, M. I. *et al.* *In Vivo* Imaging of Retinal Hypoxia in a Model of Oxygen-Induced Retinopathy. *Sci. Rep.* **6**, 31011; doi: 10.1038/srep31011 (2016).

## Supplementary Material

Supplementary Information

## References

[b1] Gariano R. F. & Gardner T. W. Retinal angiogenesis in development and disease. Nature 438, 960–966, doi: 10.1038/nature04482 (2005). 16355161

[b2] Anderson C. G., Benitz W. E. & Madan A. Retinopathy of prematurity (ROP) and pulse oximetry: A national survey of recent practices. Pediatr Res 51, 367a–367a (2002). 10.1038/sj.jp.7211067 14999216

[b3] Wang X. Q., Wang G. B. & Wang Y. Intravitreous Vascular Endothelial Growth Factor and Hypoxia-Inducible Factor 1a in Patients With Proliferative Diabetic Retinopathy. Am J Ophthalmol 148, 883–889, doi: 10.1016/j.ajo.2009.07.007 (2009). 19837381

[b4] Rehak J. & Rehak M. Branch retinal vein occlusion: Pathogenesis, visual prognosis, and treatment modalities. Curr Eye Res 33, 111–131, doi: 10.1080/02713680701851902 (2008). 18293182 PMC2430176

[b5] Hartnett M. E. & Penn J. S. Mechanisms and Management of Retinopathy of Prematurity. New Engl J Med 367, 2515–2526, doi: 10.1056/Nejmra1208129 (2012). 23268666 PMC3695731

[b6] Ashton N. & Cook C. Direct observation of the effect of oxygen on developing vessels: preliminary report. The British journal of ophthalmology 38, 433–440 (1954). 13172418 10.1136/bjo.38.7.433 PMC1324375

[b7] Ashton N. Pathological basis of retrolental fibroplasia. The British journal of ophthalmology 38, 385–396 (1954). 13172416 10.1136/bjo.38.7.385 PMC1324373

[b8] O’Mahoney P. R. A., Wong D. T. & Ray J. G. Retinal vein occlusion and traditional risk factors for atherosclerosis. Arch Ophthalmol-Chic 126, 692–699, doi: 10.1001/archopht.126.5.692 (2008). 18474782

[b9] Robbins S. G., Conaway J. R., Ford B. L., Roberto K. A. & Penn J. S. Detection of vascular endothelial growth factor (VEGF) protein in vascular and non-vascular cells of the normal and oxygen-injured rat retina. Growth Factors 14, 229-&, doi: 10.3109/08977199709021522 (1997). 9386988

[b10] Xin X. B. *et al.* Hypoxic retinal Muller cells promote vascular permeability by HIF-1-dependent up-regulation of angiopoietin-like 4. P Natl Acad Sci USA 110, E3425–E3434, doi: 10.1073/pnas.1217091110 (2013). PMC3767527 23959876

[b11] Linsenmeier R. A. *et al.* Retinal hypoxia in long-term diabetic cats. Invest Ophth Vis Sci 39, 1647–1657 (1998). 9699554

[b12] Berkowitz B. A. & Penn J. S. Abnormal panretinal response pattern to carbogen inhalation in experimental retinopathy of prematurity. Invest Ophthalmol Vis Sci 39, 840–845 (1998). 9538894

[b13] Hardarson S. H. *et al.* Automatic retinal oximetry. Invest Ophthalmol Vis Sci 47, 5011–5016, doi: 10.1167/iovs.06-0039 (2006). 17065521

[b14] Shahidi M., Shakoor A., Blair N. P., Mori M. & Shonat R. D. A method for chorioretinal oxygen tension measurement. Curr Eye Res 31, 357–366, doi: 10.1080/02713680600599446 (2006). 16603469 PMC2902869

[b15] Dai C., Liu X., Zhang H. F., Puliafito C. A. & Jiao S. Absolute retinal blood flow measurement with a dual-beam Doppler optical coherence tomography. Invest Ophthalmol Vis Sci 54, 7998–8003, doi: 10.1167/iovs.13-12318 (2013). 24222303 PMC3858018

[b16] Soetikno B. T. *et al.* Inner retinal oxygen metabolism in the 50/10 oxygen-induced retinopathy model. Scientific reports 5, 16752, doi: 10.1038/srep16752 (2015). 26576731 PMC4649746

[b17] Scott A. & Fruttiger M. Oxygen-induced retinopathy: a model for vascular pathology in the retina. Eye 24, 416–421, doi: 10.1038/eye.2009.306 (2010). 20010791

[b18] Busk M. *et al.* PET imaging of tumor hypoxia using 18F-labeled pimonidazole. Acta oncologica 52, 1300–1307, doi: 10.3109/0284186X.2013.815797 (2013). 23962243

[b19] Kizaka-Kondoh S. & Konse-Nagasawa H. Significance of nitroimidazole compounds and hypoxia-inducible factor-1 for imaging tumor hypoxia. Cancer science 100, 1366–1373, doi: 10.1111/j.1349-7006.2009.01195.x (2009). 19459851 PMC11158459

[b20] Yang Y. *et al.* Magnetic resonance imaging retinal oximetry: a quantitative physiological biomarker for early diabetic retinopathy? Diabetic medicine: a journal of the British Diabetic Association 29, 501–505, doi: 10.1111/j.1464-5491.2011.03440.x (2012). 21913965

[b21] Mowat F. M. *et al.* HIF-1alpha and HIF-2alpha are differentially activated in distinct cell populations in retinal ischaemia. PloS one 5, e11103, doi: 10.1371/journal.pone.0011103 (2010). 20559438 PMC2885428

[b22] Varia M. A. *et al.* Pimonidazole: A novel hypoxia marker for complementary study of tumor hypoxia and cell proliferation in cervical carcinoma. Gynecol Oncol 71, 270–277, doi: 10.1006/gyno.1998.5163 (1998). 9826471

[b23] Uddin M. I. *et al.* Applications of Azo-Based Probes for Imaging Retinal Hypoxia. Acs Med Chem Lett 6, 445–449, doi: 10.1021/ml5005206 (2015). 25893047 PMC4394343

[b24] Evans S. M. *et al.* Molecular Probes for Imaging of Hypoxia in the Retina. Bioconjugate Chem 25, 2030–2037, doi: 10.1021/bc500400z (2014). PMC4240343 25250692

[b25] Ross D., Beall H. D., Siegel D., Traver R. D. & Gustafson D. L. Enzymology of bioreductive drug activation. The British journal of cancer. Supplement 27, S1–S8 (1996). 8763836 PMC2150032

[b26] Limb G. A., Salt T. E., Munro P. M., Moss S. E. & Khaw P. T. *In vitro* characterization of a spontaneously immortalized human Muller cell line (MIO-M1). Invest Ophthalmol Vis Sci 43, 864–869 (2002). 11867609

[b27] Smith L. E. *et al.* Oxygen-induced retinopathy in the mouse. Invest Ophthalmol Vis Sci 35, 101–111 (1994). 7507904

[b28] Domenici L., Berardi N., Carmignoto G., Vantini G. & Maffei L. Nerve growth factor prevents the amblyopic effects of monocular deprivation. Proc Natl Acad Sci USA 88, 8811–8815 (1991). 1924342 10.1073/pnas.88.19.8811 PMC52600

[b29] Rex T. S. *et al.* Systemic but not intraocular Epo gene transfer protects the retina from light-and genetic-induced degeneration. Molecular therapy: the journal of the American Society of Gene Therapy 10, 855–861, doi: 10.1016/j.ymthe.2004.07.027 (2004). 15509503

[b30] Connor K. M. *et al.* Quantification of oxygen-induced retinopathy in the mouse: a model of vessel loss, vessel regrowth and pathological angiogenesis. Nature protocols 4, 1565–1573, doi: 10.1038/nprot.2009.187 (2009). 19816419 PMC3731997

[b31] Stahl A. *et al.* The mouse retina as an angiogenesis model. Invest Ophthalmol Vis Sci 51, 2813–2826, doi: 10.1167/iovs.10-5176 (2010). 20484600 PMC2891451

[b32] Penn J. S. *et al.* Vascular endothelial growth factor in eye disease. Prog Retin Eye Res 27, 331–371, doi: 10.1016/j.preteyeres.2008.05.001 (2008). 18653375 PMC3682685

[b33] Lau J. C. & Linsenmeier R. A. Oxygen consumption and distribution in the Long-Evans rat retina. Exp Eye Res 102, 50–58, doi: 10.1016/j.exer.2012.07.004 (2012). 22828049 PMC3437263

[b34] Yu D. Y. & Cringle S. J. Oxygen distribution in the mouse retina. Invest Ophthalmol Vis Sci 47, 1109–1112, doi: 10.1167/iovs.05-1118 (2006). 16505048

[b35] Beebe D. C., Shui Y. B., Siegfried C. J., Holekamp N. M. & Bai F. Preserve the (intraocular) environment: the importance of maintaining normal oxygen gradients in the eye. Japanese Journal of Ophthalmology 58, 225–231, doi: 10.1007/s10384-014-0318-4 (2014). 24687817

[b36] Wangsa-Wirawan N. D. & Linsenmeier R. A. Retinal oxygen: fundamental and clinical aspects. Arch Ophthalmol 121, 547–557, doi: 10.1001/archopht.121.4.547 (2003). 12695252

[b37] Ernest J. T. & Goldstick T. K. Retinal oxygen tension and oxygen reactivity in retinopathy of prematurity in kittens. Invest Ophthalmol Vis Sci 25, 1129–1134 (1984). 6207136

[b38] Eichler W., Yafai Y., Keller T., Wiedemann P. & Reichenbach A. PEDF derived from glial Muller cells: a possible regulator of retinal angiogenesis. Exp Cell Res 299, 68–78, doi: 10.1016/j.yexcr.2004.05.020 (2004). 15302574

