# Supplementary material for: In Vivo Imaging of Retinal Hypoxia in a Model of Oxygen-Induced Retinopathy
Source: Sci Rep. 2016 Aug 5;6:31011. doi: 10.1038/srep31011 (PMC4974503; doi:10.1038/srep31011)
Supplement: Supplementary Information [file srep31011-s1.pdf]

Supporting Information:

In Vivo Imaging of Retinal Hypoxia in a Model of Oxygen-Induced Retinopathy

Md. Imam Uddin,*[1] Stephanie M. Evans,[1] Jason R. Craft,[1] Megan E. Capozzi,[4] Gary W. McCollum,[1] Rong Yang,[1] Lawrence J. Marnett,[2] Md. Jashim Uddin,[2] Ashwath Jayagopal,[3] and John S. Penn,*[1,4]

Authors' Affiliations

[1] Department of Ophthalmology and Visual Sciences, Vanderbilt University School of Medicine, Nashville, TN, USA.

[2] A. B. Hancock, Jr., Memorial Laboratory for Cancer Research, Departments of Biochemistry, Chemistry and Pharmacology, Vanderbilt Institute of Chemical Biology, Center for Molecular Toxicology and Vanderbilt-Ingram Cancer Center, Vanderbilt University School of Medicine, Nashville, TN, USA.

[3] Hoffmann-La Roche Ltd, Basel, Switzerland.

[4] Department of Molecular Physiology and Biophysics, Vanderbilt University School of Medicine, Nashville, TN, USA.

Running Title: *In vivo* Imaging of Retinal Hypoxia

Key Words: *in vivo* imaging, retinal hypoxia, ischemia, oxygen-induced retinopathy

GENERAL CHEMISTRY TECHNIQUES

All chemicals were purchased and used as received unless otherwise indicated. The nitroimidazole-analog was synthesized according to a modified procedure [1, 2]. Moisture sensitive reactions were performed in oven-dried glassware under a positive pressure of nitrogen or argon. Air and moisture-sensitive compounds were introduced via syringe or cannula through a rubber septum. HPLC grade solvents were obtained from Fisher Scientific (Pittsburgh, PA). All reagents and deuterated solvents were purchased from the Aldrich Chemical Company (Milwaukee, WI) and used without further purification. The Oregon Green 488 carboxylic acid succinimidyl ester, 5-isomer was purchased from Life Technologies (Grand Island, NY) and used without further purification.

Chromatography

Silica gel column chromatography was performed using Sorbent silica gel standard grade, porosity 60 Å, particle size 32-63 (μm) (230 x 450 mesh), surface area 500-600 m^2/g, bulk density 0.4 g/mL, pH range 6.5-7.5, purchased from Sorbent Technologies (Atlanta, GA). The analytical HPLC of the fluorescent compounds were performed on a Waters 2996 HPLC system with a UV or fluorescence detector using C18 reverse-phase columns. HYPOX-4 compound used for biological assays was ≥ 95% purity based on analytical HPLC monitored at 490 nm.

Synthesis of HYPOX-4

To a stirred solution of pimonidazole amine hydrochloride (compound **3**, Scheme SI1) (28.2 μmol) in dimethylsulfoxide (2 mL) triethylamine was added (0.1 μmol) to generate the free amine. After stirring for 5 min, the Oregon Green 5-carboxylic acid succinimidyl ester, (compound **4**, Scheme SI1) (15.7 μmol) was added and stirred overnight at 25 ^oC. The solvent was removed by lyophyllization to give the crude product, which was purified by silica gel column chromatography.

Orange solid (75%). ^1H-NMR (400 MHz, DMSO-d_6) δ10.86 (bs, 1H), 9.68 (bs, 1H), 8.94 (t, J = 5.8 Hz, 1H), 8.46 (s, 1H), 8.26 (m, 1H), 7.63 (m, 1H), 7.40 (d, J = 8.0 Hz, 1H), 7.19 (m, 1H), 6.93 (m, 2H), 6.53 (m, 2H), 4.57 (dd, J = 17.2, 7.4 Hz, 1H), 4.38 (m, 2H), 3.60-3.49 (m, 4H), 3.23-3.21 (m, 2H), 3.18-3.15 (m, 1H), 3.08–2.89 (m, 2H), 1.89-1.83 (m, 2H, 1.67-1.59 (m, 1H, 1.55-1.49 (m, 1H); ^{19}F-NMR (282 MHz, DMSO-d_6) δ -139.9; Mass (ESI-) calcd for $C_{33}H_{28}F_2N_5O_9$ [M-H]: 676.6; found: 676.4.

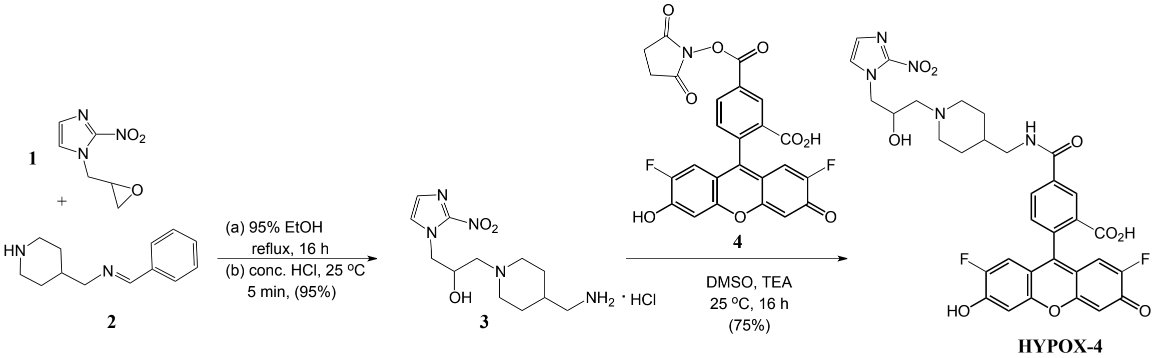

Scheme S1. Synthesis of HYPOX-4.

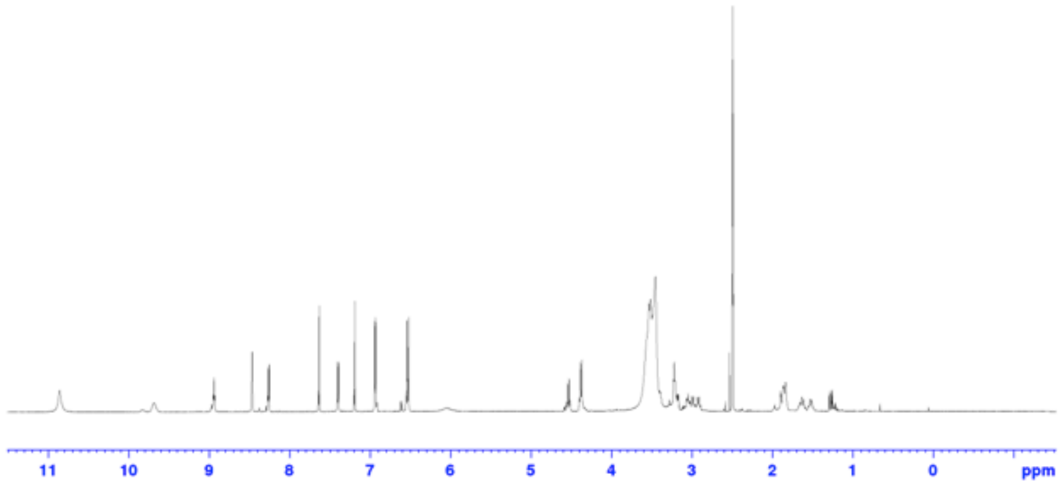

Figure S1: ^{1}H-NMR spectra of compound HYPOX-4.

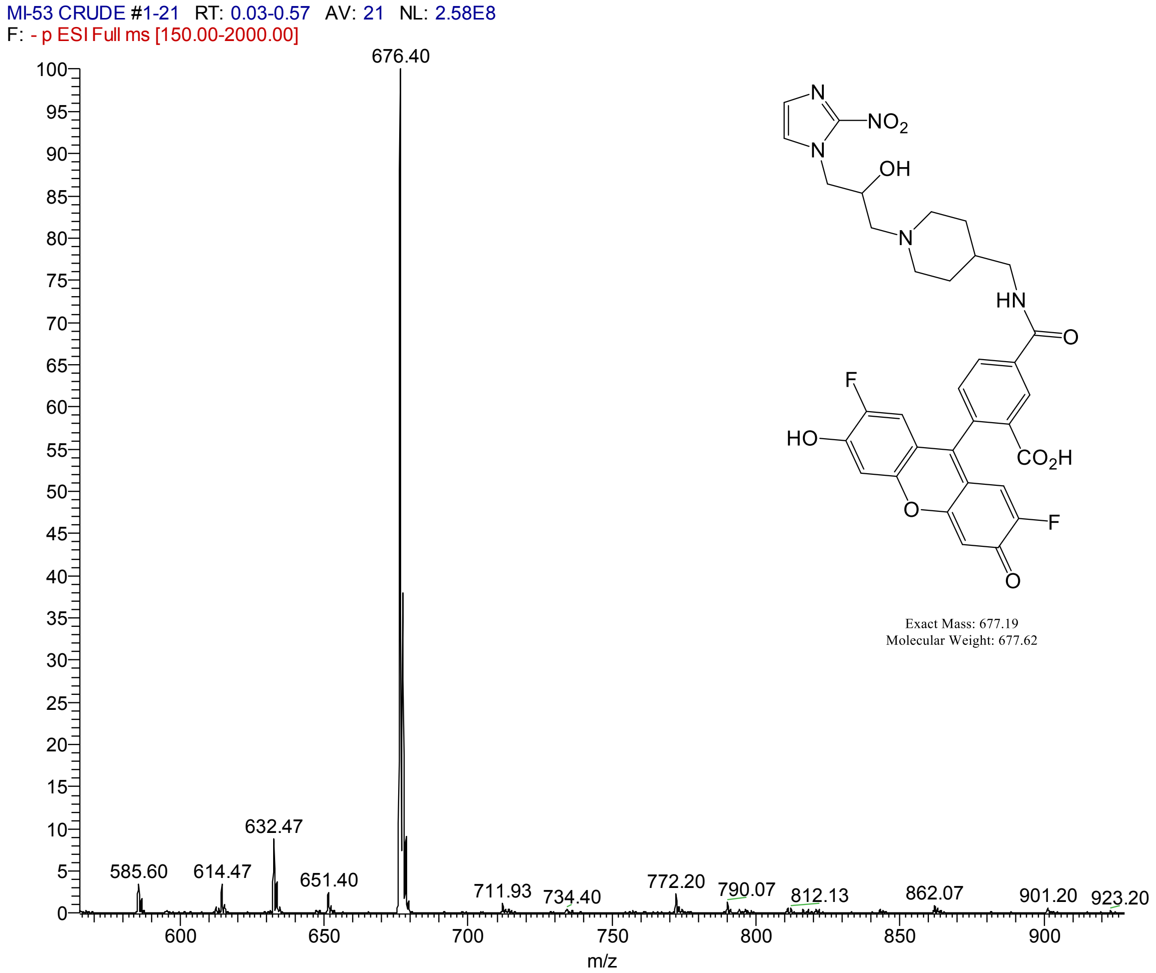

Figure S2: LRMS data of compound HYPOX-4.

Determination of octanol-water partition coefficient

HYPOX-4 (173 μg, 0.26 μmol), pimonidazole free base (64 μg, 0.26 μmol) or pimonidazole HCl (1.5 μL, 50 mg/mL) was added to a mixture of octanol (500 μL) and water (500 μL) in an eppendrof tube and gently mixed on a rotator for 2 days. The aqueous and octanol layers were separated and the optical density (OD) was measured in microplate spectrophotometer instrument. Standard curves of fluorescence vs concentration series of HYPOX-4, pimonidazole free base and pimonidazole HCl in octanol or water were generated. The quantities of each compound in the octanol and water layers were calculated by linear regression analysis.

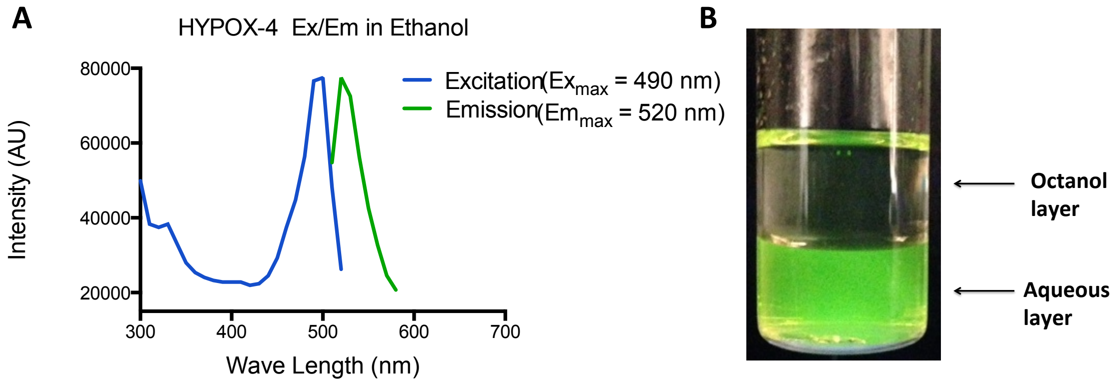

Figure S3: Photophysical properties of HYPOX-4. (A) HYPOX-4 was highly fluorescent with excitation maximum at 490 nm and emission at 520 nm. (B) The probe was also soluble at a concentration of 100 μM in aqueous solvents (neat water or saline) and almost all aqueous buffers (PBS) including cell-culture mediums (DMEM with 10%FBS).

Table S1: Solubility of HYPOX-4. The octanol-water coefficient of HYPOX-4, Pimonidazole HCl and Pimonidazole Free Base were compared.

Compounds	LogP
Pimonidazole HCl	-2.06
Pimonidazole free base	+0.74
HYPOX-4	-2.58

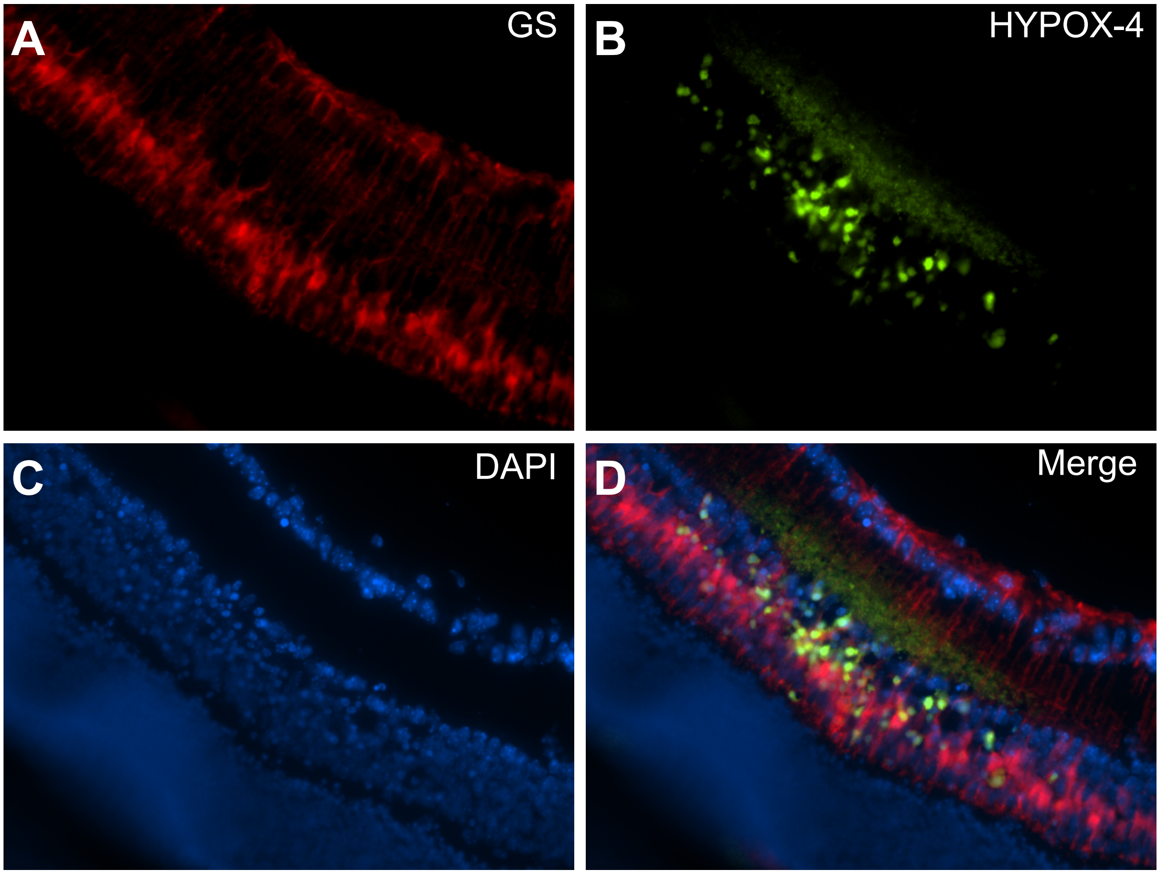

Figure S4: Immmunofluorescence staining of human Müller cells with GS (red) and HYPOX-4 (green) was localized in the inner retinal cross-sections from P13 OIR pups. DAPI (blue) represents nuclear staining. Colocalization of Müller cells staining (red) with HYPOX-4 (green) was minimally overlapped in OIR retinal cross section.

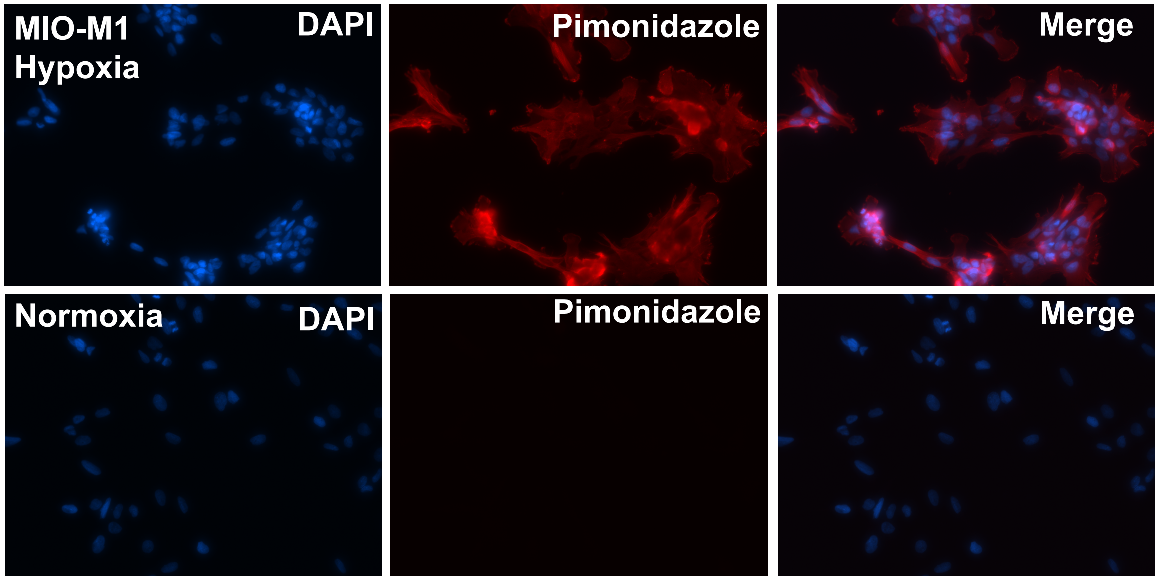

Figure S5: Pimonidazole-adduct immunodetection of hypoxia in human Müller cells (MIO-M1). Significant fluorescence enhancement was observed in the hypoxic cells incubated with Pimonidazole hydrochloride (100 μM) for 4 hours. Normoxic cells showed minimal fluorescence signal after same treatment.

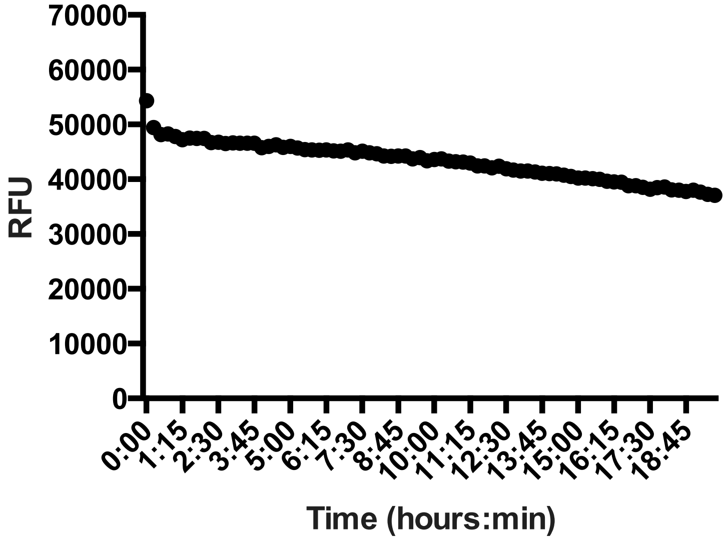

Figure S6: *In vitro* stability of HYPOX-4 in solution at 37 °C. HYPOX-4 was dissolved in phosphate buffer at a concentration of 100 nM and fluorescence was monitored at excitation maximum 490 nm and emission at 520 nm. HYPOX-4 possesses high photostability in solution for at least 20 hours.

REFERENCES

1. Okuda, K. *et al*. 2-Nitroimidazole-Tricarbocyanine Conjugate as a Near-Infrared Fluorescent Probe for in Vivo Imaging of Tumor Hypoxia. *Bioconjugate Chem* **23**, 324-329 (2012).
2. Evans, S.M. *et al*. Molecular Probes for Imaging of Hypoxia in the Retina. *Bioconjugate Chem* **25**, 2030-2037 (2014).
